# Differential Response of Mono Mac 6, BEAS-2B, and Jurkat Cells to Indoor Dust

**DOI:** 10.1289/ehp.9874

**Published:** 2007-06-01

**Authors:** Herbert Riechelmann, Tom Deutschle, Ariane Grabow, Birger Heinzow, Werner Butte, Rudolf Reiter

**Affiliations:** 1 Department of Otorhinolaryngology, University Hospital Center, Ulm, Germany; 2 Department of Environmental Health and Toxicology, State Agency for Nature and Environment of Schleswig Holstein, Kiel, Germany; 3 Faculty of Chemistry, University of Oldenburg, Oldenburg, Germany

**Keywords:** air pollution, analytical, cells, chemistry, cultured, expression profiling, gene, immunoassay, indoor dust, respiratory mucosa

## Abstract

**Background:**

Airway toxicity of indoor dust is not sufficiently understood.

**Objectives:**

Our goal in this study was to describe the effects of indoor dust on human monocyte, epithelial, and lymphocyte cell lines. We aimed to *a*) obtain a comprehensive and intelligible outline of the transcriptional response; *b*) correlate differential transcription with cellular protein secretion; *c*) identify cell line–specific features; and *d*) search for indoor dust–specific responses.

**Methods:**

Settled dust was sampled in 42 German households, and various contaminants were characterized. We exposed Mono Mac 6, BEAS-2B, and Jurkat cells to 500 μg/mL indoor dust for 6 hr. Outcome parameters included the transcriptional profile of an oligonucleotide microarray covering 1,232 genes. Significantly enriched Gene Ontology themes were calculated. Supernatant protein levels of 24 inflammatory response proteins served to confirm transcriptional results.

**Results:**

An intraclass correlation coefficient of 0.8 indicated reasonable microarray reproducibility. The transcriptional profile was characterized by enhancement of detoxification and a danger and defense response. Differential gene regulation correlated with protein secretion (Goodman and Kruskal’s gamma coefficient: 0.72; *p* < 0.01). Mono Mac 6 cells revealed the highest fraction of differentially expressed genes, dominated by up-regulation of various cytokines and chemokines. BEAS-2B cells revealed weaker changes in a limited set of inflammatory response proteins. No significant changes were observed in Jurkat cells.

**Conclusions:**

Monocytes are particularly responsive to indoor dust. We observed a classical T-helper 1-dominated immune response, which suggested that bioorganic contaminants are relevant effectors in indoor dust.

Indoor dust contributes substantially to individual particle exposure (Butte and Heinzow 2002). Compounds tracked in from outdoors are a relevant source of indoor dust contaminants. Indoor penetration from outdoors has been calculated as 70% for trace elements including various transition metals, 50% for particles, and 35% for fungal spores (Butte and Heinzow 2002; Wallace et al. 2003). Indoor dust sources include lead from paint; pyrethroids from carpets and textiles; phenols, including pentachlorophenol and bisphenol A, from wood preservatives and pesticides; organochlorines and organophosphates from pesticides; polycyclic aromatic hydrocarbons (PAHs) from heating, smoking, cooking, and parquet floor glues; phthalates from softeners used in various home products; polychlorinated biphenyls (PCBs) from plastics and sealing materials; and polybrominated diphenyl esters from flame retardants (Butte and Heinzow 2002; Wallace et al. 2003). Moreover, higher organic carbohydrate and endotoxin levels have been reported in indoor rather than in outdoor particles (Long et al. 2001; Thorne et al. 2005), and the counts of viable bacteria are apparently higher in indoor air than in outdoor air (Gorny and Dutkiewicz 2002).

Few studies have addressed effects of indoor dust on airway mucosa cells. In one study, indoor dust from two buildings was found to be a particularly strong inducer of interleukin-6 (IL-6) and IL-8 release in airway epithelial cells (Saraf et al. 1999). Similarly, Long et al. (2001) reported that indoor particles induced significantly higher tumor necrosis factor (TNF) production than outdoor particles in alveolar macrophages, even when corrected for higher indoor endotoxin concentrations. In the present study, we characterized an indoor dust sample representative for German households and investigated its effect on human airway cells. As a surrogate for cells occurring in airway mucosa, we used the monocyte cell line Mono Mac 6 (MM6), the epithelial cell line BEAS-2B (B2B), and the T-cell line Jurkat (JKT). The cellular response to indoor dust was assessed with an oligonucleotide cDNA microarray (Bammler et al. 2005). Its 1,232 genes were selected based on differential transcription of human airway mucosa specimens in whole genome arrays under various experimental conditions. On the protein level, we studied the release of various cytokines using a microsphere-based flow cytometric assay. We questioned *a*) whether an intelligible outline of cell responses to indoor dust can be obtained using gene transcription profiling; *b*) if cell line–specific differences of the transcriptional and secretory response to indoor dust can be identified; *c*) if differential gene regulation in response to indoor dust corresponds with cellular protein secretion; and *d*) whether indoor dust specific responses can be observed.

## Methods

### Indoor dust

Indoor dust was collected in 42 households in northern Germany with standard vacuum cleaners. In the ≤ 32-μm fraction of the pooled sample, we determined the concentrations of PAHs, PCBs, phthalates, organophosphates, organochlorines, phenols, pyrethroids, and metals using mass spectrometry and gas chromatography (HP G1800A, GCD Series II MS; Gen Tech Scientific, Arcade, NY, USA). Tensides (surfactants), which facilitate indoor dust absorption in a watery airway mucus blanket (Butte and Heinzow 2002), were assessed with a detergent test kit (Dr. Lange GmbH, Dusseldorf, Germany) employing sodium dodecylsulfate (anionic surfactants), Triton X-100 (non-ionic surfactants), and cetyltrimethylammonium bromide (cationic surfactants) as calibrators. For fungal spore detection, we dissolved 10 mg indoor dust in 1 mL 0.9% sodium chloride containing 0.1% Triton X-100. One hundred milliliters of this solution was then streaked on Sabouraud agar plates in 1:1, 1:10, and 1:100 dilutions; cultivated for 7 days; and visualized with Lactophenol blue. To determine total bacterial counts, 0.2133 g indoor dust was dissolved in 7 mL sterile Ringer’s solution and mounted on Columbia 5% sheep blood agar (DSMZ, Braunschweig, Germany). We assessed endotoxin concentrations using the Kinetic-QCL kit (Cambrex, Baltimore, MD, USA) according to the manufacturer’s instructions. Concentrations of several indoor allergens [i.e., those of mite (Der p1, Der f1), cat (Fel d1), and cockroach (Bla g2)] were determined with an immunodot assay (Dustscreen; CMG Heska, Fribourg, Switzerland). The presence of grass, alder, birch, and yew pollen was visualized in the dust samples by light microscopy.

### Cell cultures and protein assay

The human monocyte cell line MM6, the human T-cell line JKT (German Resource Centre for Biological Material, Braunschweig, Germany), and the human bronchial epithelial cell line B2B (Cell Concepts, Umkirch, Germany) were adjusted to 5 × 10^5^ cells/mL and grown to 80% confluence (B2B) or for a period of 24 hr (MM6 and JKT) in RPMI 1640 media supplemented with 10% fetal calf serum, 2 mM l-glutamine, 1% non-essential amino acids, and 1 mM sodium pyruvate, all from Promo Cell (Heidelberg, Germany); and 50 μg/mL penicillin and 50 μg/mL streptomycin both from Biochrom (Berlin, Germany). For each cell line, three cultures served as controls and three cultures were exposed to 500 μg/mL indoor dust for 6 hr. Cell viability was assessed before and after exposure with trypan blue dye exclusion. For gene expression analysis, RNA was extracted using the RNeasy Mini Kit (Qiagen, Cologne, Germany), and exposed cultures were compared with the corresponding control. In the supernatants, we used a microsphere-based flow cytometric assay (Luminex System; Microbionix, Munich, Germany) to detect 24 cytokines. The limits of detection (LOD) of this assay ranged between 6.9 and 14.8 pg/mL.

### Microarray spotting and hybridization

Ultra Gaps II coated slides (Corning, Schiphol-Rijk, The Netherlands) were spotted using an OmniGrid 100 spotter (Gene-machines, San Carlos, CA, USA). We applied 70mer oligonucleotides (all oligonucleotides from Operon Biotechnologies, Inc., Cologne, Germany) and controls in four repetitive spots. On each slide, we included 1,232 human genes and 10 different extrahuman spiking controls from *Arabidopsis* and *Sinorhizobium* genes. Spotting buffer [3 saline sodium citrate (3SSC ):3 M NaCl, plus 0.3 M Na citrate and 1.5 M betaine (Sigma, Deisenhofen, Germany)] and randomized negative controls (i.e., oligonucleotides that do not bind human mRNA) served as controls in 300 and 12 spot quadruples, respectively. For immobilization, oligonucleotides were incubated for 10 min at 80°C followed by ultraviolet cross-linking (Stratalinker 2400; Stratagene, La Jolla, CA, USA) with 120 mJ/cm^2^. Spotting accuracy was checked with random 9mere (Operon Biotechnologies, Inc.).

For hybridization of spiking controls, synthetic mRNA-oligonucleotides of 10 different *Arabidopsis* and *Sinorhizobium* genes were synthesized on an ABI 394 synthesizer (Applied Biosystems, Foster City, CA, USA; PURIMEX, Staufenberg, Germany). Of this spiking mRNA, 200–10,000 fg was added to 5 μg total RNA of control and exposure cultures resulting in ratios of 1:2 to 1:10. Then, 5 μg total RNA from control cultures was reverse transcribed with an oligo dT primer carrying a cDNA capture sequence for the fluorescent dye cyanine 3 (Cy3), and 5 μg total mRNA from dust-exposed cultures with a capture sequence for cyanine 5 (3 DNA Array 350 Kit; Genisphere, Hatfield, PA, USA). The resulting cDNA was further purified and concentrated with Millipore Microcon YM-30 Centrifugal Filter Device (Millipore, Billerica, MA, USA) and mounted on the spotted slides prehybridized with bovine serum albumin for 1 hr. cDNA was allowed to hybridize with the spotted oligonucleotides at 57°C overnight. cDNA hybridized with the spotted oligonucleotides was then incubated for 3 hr at 59°C with the 3DNA capture reagent.

### Microarray data analysis

**Figure 1:**
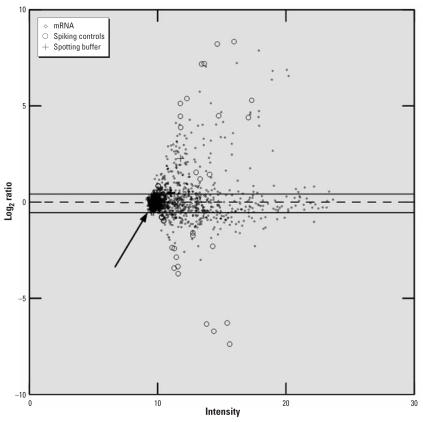
Sample ratio intensity plot in MM6 cells. The dashed line indicates the median. Upper and lower outer fences of 300 spotting-buffer quadruples (arrow) were calculated according to Tukey and Aase (1977). Oligonucleotides of 10 *Arabidopsis* and *Shinorizobium* genes were synthesized and added to the probes in concentration of 200–10,000 fg (spiking controls). Spiking controls were reproducibly separated from buffer spots, whereas randomized negative controls (i.e., oligonucleotides that do not bind human RNA) were always within the calculated fences (not visible due to high spot density). Genes (rhombi) outside the fences were regarded as differentially transcribed. The *y*-axis is the dual logarithm of mRNA spot intensity after indoor dust exposure divided by spot intensity after control exposure; the *x*-axis is the geometric mean of spot intensity (arbitrary scale).

Slides were scanned on a dual-laser microarray scanner (GenePix 4000 B; Axon Instruments, Foster City, CA) and analyzed with Gene Pix pro 4.1 software (Axon Instruments, Foster City, CA, USA). We used a nonparametric algorithm without background subtraction to assess differential gene expression. In a recent report (Bammler et al. 2005), background subtraction was identified as a significant source of data variability. Therefore, instead of background subtraction, we disregarded spots with a background above the 99th percentile. From the remaining spots per gene, we calculated the median intensity if at least three of four repetitive spots were available. Following lowess- and block-normalization, the dual logarithm of the Cy5/Cy3 intensity ratio was formed for all genes and controls. To identify differentially regulated genes, we calculated the upper and lower quartiles of the log_2_ intensity ratios of the 300 control spot quadruples (spotting buffer only). Following the method of Tukey and Aase (1977), we calculated outer fences three interquartile ranges above or below the hinges of the control spots. Values outside the outer fences of the log_2_ ratios of the 300 control spots were defined as differentially transcribed. With this algorithm, control spots could be reproducibly separated from spiking controls (Figure 1). For each cell line, only genes up- or down-regulated in at least two of three arrays were considered differentially transcribed. All calculations were performed with Systat 10.2 (Systat Software Inc., Richmond, CA, USA).

### Gene-annotation enrichment analysis

Genes were named according to the Human Genome Organization and grouped into categories defined by the Gene Ontology (GO) Consortium (Bammler et al. 2005; GO Consortium 2006) based on their molecular function and the involved biological process. The number of observed versus expected differentially transcribed genes per GO category were calculated using the web-based platform GOTree Machine (Zhang et al. 2004). In this context, the expected number of differentially transcribed genes equals the number per GO category, if all categories on the particular array are equally affected by up- or down-regulation. The expected number thus represents the fraction of differentially transcribed genes on the whole array multiplied by the number of genes in a particular GO-category on this array, and is not directly associated with control exposure. The enrichment factor equals the odds ratio of expected and actually observed differentially transcribed genes per GO category. We present only significantly enriched GO categories containing a disproportionate amount of differentially transcribed genes (Fisher’s exact *p* < 0.01). These categories provide detailed, cell line–specific information about transcriptional responses to indoor dust. To provide an intelligible overview of biological processes, we used 14 GO slim terms defined by the GO Consortium (Harris et al. 2004). GO slims are a reduced version of the GO ontologies representing a high-level summary of molecular functions and biological processes of differentially transcribed genes. They are presented for all three cell lines, whether enriched or not, and thus allow a direct comparison of the responses of the three cell lines.

## Results

### Indoor dust

We grouped indoor dust contaminants according to chemical and functional characteristics (Table 1). Most contaminants were within 95% percentiles of dusts previously reported in German households (Butte and Heinzow 2002). Copper concentrations were slightly above the 95th percentile and lead concentrations were near the 90th percentile. The concentrations of germinable fungal spores were 80.00 ± 7.500 colony forming units (CFU)/g dust (mean ± SD), with *Penicillium* spp. (72.000 ± 6.200 CFU) dominating over *Aspergillus* spp. (8.000 ± 720 CFU). The total concentration of bacteria was 1.1 × 10^6^ ± 0.5 × 10^5^ CFU/g dust, and endotoxin activity was 15.8 ± 0.6 endotoxin units/g. Concentrations of Der p1 and Der f1 were each 2 ± 0.1 μg/g, and those of Fel d1 and Bla g2 were 5.4 ± 0.5 μg/g and < 0.15 ± 0.01 μg/g, respectively. Pollen from weeds, alder, birch, and yew were identified but not quantified.

### Cell exposure and RNA extraction

Cell concentrations in the various culture experiments were comparable, ranging between 1.2 ± 0.1 × 10^6^ and 1.3 ± 0.2 × 10^6^ cells/mL (mean ± SD). Exposure to indoor dust did not result in relevant cell death. After exposure to indoor dust, 95 ± 2% (mean ± SD) of MM6 cells were viable (control, 96 ± 1.9%), as were 85 ± 5% of B2B cells (control, 89 ± 4%) and 95 ± 2% of JKT-cells (control, 97 ± 2%). Amounts of extracted total RNA from dust-exposed cells were slightly higher than after control exposure. The amount of extracted total RNA after dust exposure was 15.7 ± 3.3 μg versus 14.8 ± 2.3 μg after control exposure in MM6 cells, 19.7 ± 5.1 μg (exposure) versus 18.8 ± 4.2 μg (control) in B2B cells, and 12.7 ± 3.4 μg (exposure) versus 11.8 ± 1.3 μg (control) in JKT cells.

### Microarray reproducibility

**Figure 2:**
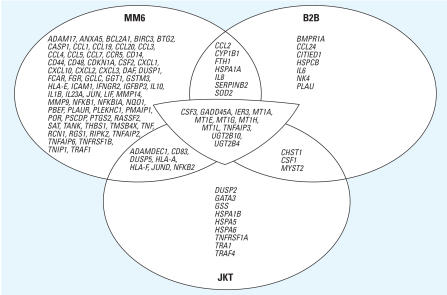
Venn diagram of up-regulated genes in MM6, B2B, and JKT cells after 6-hr exposure to 500 μg/mL indoor dust. An annotated list of up- and down-regulated genes is available in the Supplemental Material (http://www.ehponline.org/docs/2007/9874/suppl.pdf).

The three arrays per cell line yielded reasonably consistent results. The intraclass correlation coefficients of the log_2_ ratios between the three arrays per cell type were 0.87 (0.86–0.88, 95% confidence interval) for MM6, and 0.79 (0.78–0.80) for both B2B and JKT. In none of the nine arrays were the 12 randomized negative controls categorized as differentially transcribed. In contrast, all 10 spiking controls were grouped as differentially transcribed in all arrays. The log_2_ intensity ratios of the spiking controls compared well among the nine arrays, with an average coefficient of variation of 0.32. MM6 cells revealed the highest number of up-regulated genes (*n* = 90), followed by B2B (*n* = 28) and JKT cells (*n* = 30, Figure 2). In MM6 cells, 91 genes were down-regulated, whereas only 1 gene was down-regulated in B2B cells and 2 genes in JKT cells.

### Significantly enriched GO categories

Eleven genes were up-regulated in all three cell lines following exposure to house dust. Significantly enriched GO categories in all three cell lines included transition metal ion binding (observed, 5; expected, 1.21; enrichment factor, 4.13; *p* = 0.004) and UDP-glycosyltransferase activity (observed, 2; expected, 0.04; enrichment factor, 50; *p* < 0.001).

**Figure 3:**
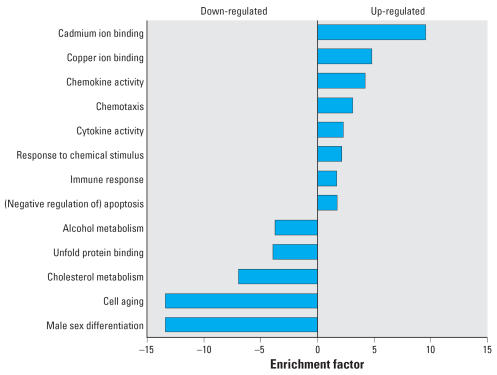
Significantly enriched GO categories (*p* < 0.01) in the domains Biological Process and Molecular Function in MM6 cells after 6 hr indoor dust exposure. The enrichment factor equals the odds ratio of the observed number divided by the expected number of up- or down-regulated genes in each GO category. For all, *p* < 0.01.

In MM6 cells, up-regulated genes were significantly overrepresented in the GO categories of cadmium and copper ion binding, chemokine and cytokine activity, and response to chemical stimulus. Moreover, the GO category apoptosis was enriched, mainly with antiapoptotic genes. Down-regulated genes were overrepresented in the GO categories alcohol and cholesterol metabolism, unfolded protein binding, cell aging, and male sex differentiation (Figure 3).

In B2B cells, up-regulated genes were significantly overrepresented in the GO categories response to chemical stimulus (enrichment factor, 3.19; *p* < 0.001), response to unfolded protein (enrichment factor, 7.69; *p* = 0.006), transition metal ion binding (enrichment factor, 2.55; *p* = 0.003), interleukin-6 receptor binding (enrichment factor, 40; *p* < 0.001), plasminogen activator activity (enrichment factor, 28.57; *p* = 0.002), and UDP-glycosyltransferase activity (enrichment factor, 13.33; *p* = 0.008). One gene, interferon regulatory factor 7 [*IRF7;* GenBank accession no. NM_004031 (Pruitt et al. 2005)] was down-regulated.

In JKT cells, significantly enriched GO categories included antiapoptosis (enrichment factor, 3.65; *p* = 0.01), transition metal ion binding (enrichment factor, 2.37; *p* = 0.005), cadmium ion binding (enrichment factor, 30.77; *p* < 0.001), copper ion binding (enrichment factor, 19.23; *p* < 0.001), zinc ion binding (enrichment factor, 3.58; *p* < 0.001), protein tyrosine/serine/threonine phosphatase activity (enrichment factor, 15.38; *p* = 0.006), MAP kinase phosphatase activity (enrichment factor, 25; *p* = 0.002), and UDP-glycosyltransferase activity (enrichment factor, 12.5; *p* = 0.009). Two genes, 3-hydroxy-3-methyl-glutaryl-coenzyme A synthase 1 (*HMGCS1;* GenBank accession no. NM_002130) and tubulin, alpha 1b (*K-ALPHA-1* or *TUBA1B*; GenBank accession no. NM_006082), were down-regulated.

### GO slim categories

**Figure 4:**
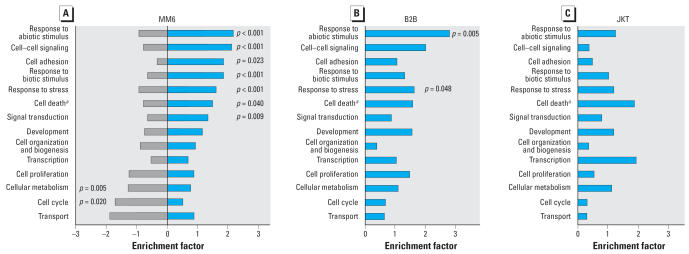
Enrichment factors of 14 GO slim categories in the domain Biological Process, representing high-level biological themes in MM6 (*A*), B2B (*B*), and JKT (*C*) cells after 6 hr indoor dust exposure. In B2B (*B*) and JKT (*C*) cells, too few genes were down-regulated for calculation of enrichment factors of down-regulated genes. ^***a***^Preferentially negative regulation of cell death.

External stimulus–related GO slim categories, including response to abiotic stimulus and response to stress, were significantly enriched in both MM6 and B2B cells. In MM6 cells, additional immune response–related GO slim categories were enriched. These included response to biotic stimulus (38 vs. 21 expected; *p* < 0.001), cell adhesion (up-regulated,12; expected, 5; *p* < 0.05) and cell–cell signaling (up-regulated, 18; expected, 9; *p* = 0.001). GO slim categories with enrichment of up-regulated genes were associated with underrepresentation of down-regulated genes, and vice versa in MM6 cells (Figure 4). GO slim categories related to external stimuli or immune responses were not significantly enriched in JKT cells.

The GO slim term “cell death” is mainly determined by programmed cell death and apoptosis. In MM6 cells, 19 cell death–related genes were up-regulated (vs. 13 expected; *p* < 0.05). However, consistent with regulation of apoptosis in all three cell lines, anti-apoptotic genes were preferentially up-regulated (observed, 9; expected, 4; *p* = 0.015). Apoptosis-related genes were not significantly enriched in B2B or JKT cells.

The GO slim terms “cell cycle” and “proliferation” relate to genes involved in cell replication and multiplication. Of the MM6 genes belonging to the GO term cell cycle, 6 were up-regulated versus 10 expected. Of these 6 genes, 4 coded for proteins with negative regulation of cell cycle. Consistently, down-regulated genes were overrepresented in the “cell cycle” category in MM6 cells (down-regulated, 15; expected, 9; *p* = 0.02). Neither slim category was significantly enriched with up- or down-regulated genes in B2B or JKT cells.

The GO slim term “signal transduction” refers to the cascade of processes mediating a change in the functioning of the cell when a receptor is activated. In MM6 cells, up-regulated genes were significantly enriched (up-regulated, 42; expected, 32; *p* = 0.009). “Transcription” in this context refers the regulation of the synthesis of RNA on a template of DNA. In neither cell line was the category “transcription” significantly enriched with up-or down-regulated genes.

The GO slim term “cellular metabolism” covers the chemical reactions and pathways by which individual cells transform chemical substances. Down-regulated genes were significantly enriched in this GO slim category (down-regulated, 49; expected, 38; *p* = 0.005) in MM6 cells. Neither up- nor down-regulated genes were significantly enriched in the GO slim categories development, cell organization and biogenesis, and transport. Although not significant, down-regulated genes revealed a comparatively high enrichment factor (1.9) in the category “transport” in MM6 cells.

### Protein concentrations

Supernatant concentrations of 24 proteins were assessed in the three cell lines exposed to indoor dust or control medium for 6 hr. The means ± SEs of three cultures per cell line per exposure group were calculated (Table 2).

Under control and exposure conditions, MM6 cells released the highest amount of cytokines. Increased cytokine release was concordant with gene up-regulation for C-C chemokine ligand 3 (CCL3), colony-stimulating factor 2 (CSF2), C-X-C chemokine ligand 10 (CXCL10), IL-1β, IL-8, and TNF-α. Increased protein release without gene up-regulation was observed for interferon gamma (IFN-γ), IL-3, and IL-6. Decreased protein concentration without gene down-regulation was observed for CCL, CCL11, IL-1α, and vascular endothelial growth factor (VEGF). No change in protein and mRNA expression was concordantly found in the remainder.

In B2B supernatants, fewer cytokines were detectable than in MM6 supernatants, and the concentrations were generally lower (Table 2). Higher cytokine concentrations after dust exposure were concordant with gene up-regulation for CCL2, IL-6, and IL-8. Higher protein concentrations without gene up-regulation were observed for IFN-γ, IL-1α, IL-3, and IL-7. Protein and mRNA expression for IL12p40 were discordant. No change in protein and mRNA expression was concordantly found for CCL5, CCL11, CXCL10, and VEGF. No differential gene regulation was observed in proteins undetectable in the B2B supernatants.

Only a few cytokines were detectable in the supernatants of JKT-cells. IL-1α supernatant concentrations were higher after dust exposure without detectable differential gene regulation. CCL11, CXCL10, and VEGF were detectable, but neither supernatant concentrations nor mRNA expression differed between control and dust exposures.

## Discussion

In this explorative investigation, we assessed short-term transcriptional and secretory responses of MM6 and B2B human cell lines to an indoor dust sample typical for German homes *in vitro*. These two cell lines frequently serve as a surrogate for airway mucosa cells. In addition, the undifferentiated human T-cell line JKT was exploratively included. Based on physiologic function of these cell lines, we hypothesized a different response pattern of the three cell lines employed. Moreover, we were interested in the correlation of the transcriptional and secretory response. The study was not designed and the results do not allow to infer on real life conditions; particularly chronic effects that are very important for human health *in vivo* were not assessed. Outcome parameters included cell viability, a transcriptional profile of 1,232 genes, and the supernatant concentrations of several cytokines and chemokines.

### Indoor dust and exposure conditions

Characteristics of dust samples from German homes were recently described by Butte and Heinzow (2002). The collected indoor dust from 42 German homes provided a reasonably representative sample. With the exception of copper, which was slightly above the 95th percentile of German indoor dust samples, contaminant concentrations were within the normal range. The selected coarse particle fraction of < 32 μm reflects the authors’ interest in the upper airways, where most particles of 10–32 μm are deposited (Keck et al. 2000). Coarse dust fractions have a higher bioorganic and lower metal content than fine fractions (Hetland et al. 2005). In the present study, we chose a dust concentration of 500 μg/mL to allow comparison with previous publications (Fujii et al. 2001; Hetland et al. 2005; Mahadevan et al. 2005) and to provide an effective cellular stimulus while leaving cells alive. In previous time-kinetic experiments, an exposure time of 6 hr revealed the highest number of transcriptional and secretory responses compared with 2 hr and 24 hr exposure. However, the transcriptional response is time dependent, and different results may be achieved at other time points.

The PAH concentrations resembled the levels of dust samples collected in home environments in the United States. Conversely, the concentrations of the synthetic pyrethroid permethrin; the phthalates benzyl butyl phthalate and di-*n*-butyl phthalate; and the phenols bisphenol A, pentachlorophenol, and nonylphenol were considerably higher than reported for the United States (Morgan et al. 2004). Chlordane, frequently detected in U.S. indoor dust, was not found. The number of germinable fungal spores was comparable with the findings in two recent studies (Gorny and Dutkiewicz 2002; Jacob et al. 2002); the number of bacteria was also comparable (Gorny and Dutkiewicz 2002). Endotoxin levels resembled concentrations found in a recent European study (Bouillard et al. 2006) and were slightly lower than in a recent investigation in the United States (Thorne et al. 2005). The content of major allergens was representative for dust samples from German cities, but considerable geographic and seasonal variations exist.

### Microarray analysis and gene-annotation enrichment analysis

One question posed by this investigation was whether plausible and interpretable transcriptional profiles can be obtained using large-scale cDNA microarrays. Interarray correlation coefficients of approximately 0.8 indicate the sound reliability of the laboratory processes (Bammler et al. 2005). The accuracy in differentiating randomized negative controls from *Arabidopsis* and *Sinorhizobium* spiking controls suggests adequate validity. The fundamental framework for the biological interpretation of gene transcription data was provided by the GO Consortium (2006). Current computational tools, such as the GOTree Machine platform, allow identification of overrepresentation of up- or down-regulated genes within the various GO categories (Zhang et al. 2004). Using these resources, we analyzed differentially transcribed genes in a two-step approach. First, GO themes significantly enriched with up- or down-regulated genes were listed including all levels of the GO domains Biological Process and Molecular Function. Thus, a comprehensive and detailed view on the transcriptional response was obtained (Figure 3). In addition, a concise transcriptional profile was obtained using a fixed set of GO slim categories (Harris et al. 2004), which allow direct comparisons of the transcriptional response of the three cell lines (Figure 4). Combining both approaches, a biologically sound profile of the cellular responses to indoor dust could be conceptualized. This transcriptional profile is characterized by enhancement of detoxification, a danger and defense response, securing of cell survival, and a cellular energy-saving program with reduced proliferative and metabolic activity.

Enhancement of detoxification was observed in all three cell lines. The metallothioneine (*MT*) genes *1A* [GenBank accession no. NM_005946.2 (Pruitt et al. 2005)], *1E* (GenBank accession no. NM_175617.3), *1G* (GenBank accession no. NM_005950.1), *1H* (GenBank accession no. NM_005951.1), and *1L* (GenBank accession no. NM_002450) were highly up-regulated. They are involved in metal detoxification and oxygen scavenging (Zhang et al. 2003). *UGT2B4* (UDP glycosyltransferase 2 family, polypeptide; GenBank accession no. NM_021139.1) and *UGT2B10* (UDP glycosyltransferase 2 family, polypeptide; GenBank accession no. NM_001075.2) belong to genes coding for uridine diphosphate glycosyltransferases involved in the conjugation and subsequent elimination of potentially toxic xenobiotics (Green and Tephly 1998). In two of the three cell lines, various additional genes involved in detoxification were up-regulated, including cytochrome P450 B1 (*CYP1B1*; GenBank accession no. NM_000104.2), superoxide dismutase 2 (*SOD2*; GenBank accession no. NM_000636.2), and ferritin heavy polypeptide 1 (*FTH1*; GenBank accession no. NM_002204.1). Interestingly, the GO term “response to oxidative stress” was not significantly enriched. In MM6 cells, we observed three up-regulated genes compared with one expected (*p* = 0.08). On closer observation, more than three genes involved in detoxification of oxygen radicals were up-regulated, including *SOD2*, NAD(P)H dehydrogenase quinone 1 [*NQO1*; GenBank accession no. NM_001025434.1 (Pruitt et al. 2005)], dual specificity phosphatase 1 (*DUSP1*; GenBank accession no. NM_004417.2) and 5 (*DUSP5*; GenBank accession no. NM_004419.3) and cytochrome P450 oxido-reductases (*POR*; GenBank accession no. NM_000941.2), which are in part regulated by Nrf2 (Li et al. 2004). Thus, a weak response to oxidative stress appears likely, which did not reveal as significantly enriched GO theme.

We observed danger and defense responses mainly in MM6 cells, and to a lower extent in B2B cells. These responses included response to stress, cell–cell signaling, and cell adhesion. Up-regulated genes included monocyte chemotactic protein 1 [*MCP-1*; GenBank accession no. NM_172361 (Pruitt et al. 2005)], *CCL2* (small inducible cytokine A2 precursor; GenBank accession no. NM_002982.3), *CCL3* (small inducible cytokine A3 precursor; GenBank accession no. NM_2983.1), *CCL4* (small inducible cytokine A4 precursor; GenBank accession no. NM_002984.2), *IL-1*β (GenBank accession no. NM_000576), *IL-6* (GenBank accession no. NM_000600), *IL-8* (GenBank accession no. NM_000584), and *TNF-*α (GenBank accession no. NM_006290). This profile is consistent with the transcriptional response of monocytes/macrophages to various stimuli including bacteria and fungi (Blumenthal et al. 2005; Cortez et al. 2006), metals (Jost-Albrecht and Hofstetter 2006), diesel exhaust particles (Li et al. 2004), and cigarette smoke (van Leeuwen et al. 2005), but differed from the transcription profile of IL-13–stimulated monocytes (Scotton et al. 2005). The response of MM6 cells thus resembles “classical” macrophage activation favoring a T-helper 1 (T_H1_)-skewed immune response (Mosser 2003).

Cell survival rather than apoptosis was promoted in all three cell lines. In MM6 cells, 19 cell death related genes were up-regulated compared with 13 expected (*p* = 0.04). Consistent with the viability assays, which did not show relevant cell death in response to indoor dust exposure, antiapoptotic genes were preferentially up-regulated.

Various transcriptional responses resembled a cellular economy drive under stress conditions. Cell functions not involved in detoxification, defense, and survival were down-regulated. Genes grouped under the GO category “cell proliferation” preferentially transmitted negative regulation of cell proliferation (*p* = 0.04). Similarly, emissions of indoor dust and ambient particulates reduced epithelial cell proliferation in two recent studies (Baulig et al. 2003; Mathiesen et al. 2004). Consistently, genes promoting the progression through the cell cycle were significantly down-regulated in MM6 cells. Down-regulated genes were also overrepresented in the GO slim categories “cellular metabolism” and “transport’” A term related to transport is “endocytosis,” including phagocytosis, which is particularly interesting in dust-exposed cells. Up- and down-regulated genes were not significantly enriched in the category “endocytosis” or in any of its related terms. This observation is in line with a recent publication, which reported decreased phagocytic activity after particle exposure (Lundborg et al. 2006).

### Cellular mRNA levels and protein secretion

In large-scale studies, microarrays demonstrated reasonable validity to assess cellular mRNA levels when compared with various polymerase chain reaction (PCR) methods (Canales et al. 2006; Cortez et al. 2006; Wang et al. 2006). In the present study microarrays were calibrated with quantitatively added mRNA spiking controls, as described by Wang et al. (2003). Moreover, we assessed the differential transcription of functional groups of genes, rendering single gene products less relevant. Additional confirmation of differential gene expression with PCR-techniques was therefore considered dispensable. However, conclusions on the transcriptional response of single genes should be made with caution without PCR confirmation, particularly if the transcriptional and secretory response are inconsistent.

In the present study we focused on the concordance of mRNA and protein expression. The correlation of cellular mRNA concentrations and protein expression depends on various parameters, including intracellular mRNA amount and location, translational regulation, ribosome density and occupancy, cellular protein degradation, experimental conditions, and stochastic noise inherent in large-scale experiments. In comparisons of mRNA levels with limited protein expression data, fair correlations were found (Andrew et al. 2003; Cortez et al. 2006; Greenbaum et al. 2003). However, on a genomic scale, mRNA–protein correlations were poor (Greenbaum et al. 2003). In the present study, we compared changes of cellular mRNA levels of 24 inflammatory response proteins with the according change in supernatant protein concentrations. Differential expression of mRNA and protein expression agreed in 53 of 69 pairs assessed (Table 3), indicating a relevant association of the transcriptional response and protein secretion in this small set of functionally corresponding proteins. Interestingly, all genes that were up-regulated at least 2-fold also showed increased protein secretion. Moreover, a T_H1_-skewed immune response profile was observed on both the transcriptional and the protein levels.

### Cell line–specific differences in the response to indoor dust

Airway mucosa contains various cell types that may differ in their response to inhaled dust constituents. One question posed by this study was whether cell line–specific differences of the transcriptional and secretory response to indoor dust can be identified. MM6 cells revealed the highest fraction of differentially transcribed defense genes and the highest supernatant concentrations of cytokines and chemokines. This suggests a key role in the mucosal defense response. The high responsiveness of MM6 cells is consistent with the physiologic function of monocytes/ macrophages representing the first line defense and surveillance of the innate immune system. Moreover, these cells are able to present antigen to immune cells, which is associated with extensive transcriptional and secretory activity.

In contrast, the main physiologic function of epithelial cells is to form a dermal or mucosal barrier. Consistent with this function, less intense transcriptional and secretory response was expected. However, epithelial cells also contribute actively to innate immune functions (Bals and Hiemstra 2004; Baulig et al. 2003). Although the result of the present study suggests that epithelial immune functions are less intense than those of macrophages, the high number of epithelial cells in the airways may render the epithelial innate immune response highly effective.

Naive T cells in respiratory mucosa are generally activated by antigen on major histo-compatibility class II (MHC-II) molecules (Del Prete et al. 1993). They are not able to phagocytize. In monoculture, JKT cells responded to a limited number of stimuli including phorbol ester, phytohemagglutinin, metals, formaldehyde, calcium ionophores, and pyrene, but not to hydrogen peroxide or mitomycin C ( Brundage et al. 2004; Saito et al. 2005; Verstraeten 2006). After exposure to indoor dust, JKT cells remained more or less immunologically dormant. This result suggests that without the help of antigen-presenting cells, indoor dust is not able to elicit a significant response in JKT cells. In coculture with MHC-matched antigen-presenting cells, JKT cells may be more intensively activated.

## Conclusion

We obtained a biologically plausible transcriptional profile in response to coarse indoor dust as deposited in the upper airways using cDNA microarrays and gene-annotation enrichment analysis. Validity of this new type of bio-informatics analysis will probably be further substantiated within the next few years. MM6, B2B, and JKT cells responded similarly with detoxification, antiapoptosis, and reduced mitotic and metabolic activity to a representative indoor dust from German homes. The three cell lines differed particularly in the intensity of their defense response on both the transcriptional and secretory levels. Consistent with previous studies, the observed defense response to indoor dust in MM6 and B2B cells promoted a T_H1_-skew and suggested weak oxidative stress and subtle DNA damage (Allermann et al. 2003; Long et al. 2001; Saraf et al. 1999). This points toward a major role of bioorganic dust constituents such as endotoxin, fungal, and bacterial contaminants. However, ambient air particles of smaller particle sizes with higher metal and lower bio-organic content induced a marked release of inflammatory mediators in association with a more pronounced oxidative stress response (Ghio 2004; Hetland et al. 2005). Also, allergens in indoor dust may alter cell functions because of their enzymatic and toxic activity (Martinez et al. 1999); however, in contrast with the *in vivo* situation, IgE mediated effects are considered less likely because mast cells were absent in the cell cultures.

## Figures and Tables

**Table 1:** Inorganic and organic compounds in indoor dust.

Compound	Concentration (ng/g)
PAHs^a^	
Benzo(*a*)anthracene	270 ± 43
Chrysene	220 ± 55
Fluoranthene	460 ± 33
Pyrene	340 ± 48
PCB congeners^b^	
PCB-101	45.6 ± 3.9
PCB-138	91.8 ± 8.2
PCB-153	69.6 ± 5.2
PCB-180	66.1 ± 4.7
Phthalates	
Benzyl butyl phthalate	34,000 ± 4,050
Di-*n*-butyl phthalate	49,200 ± 5,700
Di(2-ethylhexyl) phthalate	410
Di-iso-butyl phthalate	31,700 ± 3,360
Diethyl phthalate	44,500 ± 4,170
Pyrethoids^c^	
*cis*- and *trans*-permethrin	4,820 ± 85
Organochlorines	
γ-Hexachlorocyclohexane (lindane)	200 ± 28
*p,p*′-DDT	340 ± 28
Phenols^d^	
Bisphenol A	6,070 ± 500
Nonylphenol	11,700 ± 920
Pentachlorophenol	780 ± 30
Tensides (surfactants)	
Anionic	210,000
Non-ionic	145,000
Kationic	1,400
Metals	
Cadmium	2,870 ± 210
Chromium	117,000 ± 14,900
Copper	429,000 ± –35,000
Mercury	2,420 ± 950
Nickel	61,700 ± 3,130
Lead	174,000 ± 13,700
Pesticide synergists	
Piperonyl butoxide	600 ± 52
Organophosphates^e^	
TBEP	14,100 ± 1,140
TCEP	1,760 ± 270

Abbreviations: TBEP, tris-(2-butoxyethylester) phosphate; TCEP, tris-(2-chlorethyl) phosphate. Values are mean ± SD of six measurements except where indicated.

a Limit of detection = 100 ng/g; not detected: acenaphthylene, acenaphthene, fluorene, phenanthrene, anthracene, benzo(*b*)fluoranthene/benzo(*k*)fluoranthene, benzo(*a*)pyrene, indeno(1,2,3,*c,d*) pyrene, dibenz(*a,h*)anthracene, and benzo(*g,h,i*)perylene.

b Limit of detection = 10 ng/g; not detected: PCB-28 and PCB-52.

c Limit of detection = 100 ng/g; not detected: tetramethrin and propoxur.

d Limit of detection = 50 ng/g; not detected: various trichlorophenols and tetrachlorophenols.

e Limit of detection = 100 ng/g; not detected: triphenylphosphate, tris-(2-ethylhexyl)-phosphate, and tricresylphosphate.

**Table 2:** Cytokine concentrations (pg/mL) in the supernatants of MM6, B2B, and JKT cells.

	MM6		B2B		JKT	
	Control	Exposed	Control	Exposed	Control	Exposed
CCL2^a^	5,150 ± 5	3,926 ± 251	166 ± 35	943 ± 4	< LOD	< LOD
CCL3^b^	1,167 ± 86	3,250 ± 123	< LOD	< LOD	< LOD	< LOD
CCL5^c^	1,180 ± 0	792 ± 156	42 ± 2	33 ± 20	11 ± 1	10 ± 2
CCL11^d^	182 ± 1	109 ± 4	30 ± 7	26 ± 5	23 ± 2	23 ± 6
CSF-2^e^	8 ± 0	76 ± 2	8 ± 0	19 ± 1	< LOD	< LOD
CXCL10^f^	48 ± 7	991 ± 141	85 ± 33	92 ± 22	52 ± 4	44 ± 8
FLT3 ligand	< LOD	< LOD	< LOD	< LOD	< LOD	< LOD
IFN-γ	83 ± 3	104 ± 5	14 ± 0	38 ± 13	< LOD	< LOD
IL-1α	1,253 ± 57	389 ± 24	10 ± 1	72 ± 11	9 ± 1	85 ± 32
IL-1β	8 ± 1	65 ± 2	< LOD	< LOD	< LOD	< LOD
IL-2	< LOD	< LOD	< LOD	< LOD	< LOD	< LOD
IL-3	82 ± 1	190 ± 7	15 ± 3	94 ± 1	< LOD	< LOD
IL-4	< LOD	< LOD	< LOD	< LOD	< LOD	< LOD
IL-5	< LOD	< LOD	< LOD	< LOD	< LOD	< LOD
IL-6	43 ± 2	3,716 ± 968	111 ± 66	2,770 ± 205	< LOD	< LOD
IL-7	228 ± 4	275 ± 6	23 ± 2	93 ± 18	< LOD	< LOD
IL-8	2,553 ± 32	3,207 ± 317	57 ± 25	339 ± 39	< LOD	< LOD
IL-10	17 ± 4	14 ± 2	< LOD	< LOD	< LOD	< LOD
IL-12p40	354 ± 5	348 ± 13	35 ± 9	22 ± 4	< LOD	< LOD
IL-12p70	< LOD	< LOD	< LOD	< LOD	< LOD	< LOD
IL-13	< LOD	< LOD	< LOD	< LOD	< LOD	< LOD
IL-15	< LOD	< LOD	< LOD	< LOD	< LOD	< LOD
TNF-α	10 ± 0	2,010 ± 103	< LOD	< LOD	< LOD	< LOD
VEGF	333 ± 46	14 ± 0	73 ± 13	67 ± 10	41 ± 26	32 ± 9

a CCL2 = MCP-1.

b CCL3 = MIP1-α.

c CCL5 = RANTES.

d CCL11 = eotaxin.

e CSF-2 = GM-CSF.

f CXCL10 = IP10.

**Table 3:** Concordance of differential transcription and protein secretion.

	Supernatant protein level after dust exposure^a^		
Transcription^b^	> 25% increase	± 25%	> 25% decrease
Up-regulated	9	2	1
Not differentially regulated	9	44	4
Down-regulated	0	0	0

Goodman and Kruskal’s gamma coefficient: 0.72 (*p* < 0.01).

a Compared with control exposure.

b IL-12p70 was omitted as its levels are regulated by two genes, *IL12A* and *IL12B*.
